# Ischemic Stroke Associated With Vertebral Artery Occlusion Following Cervical Spine Manipulation in a Young Woman With Underlying Vascular Risk Factors: A Case Report

**DOI:** 10.1002/ccr3.73772

**Published:** 2026-10-20

**Authors:** Aguasanta González‐Delatorre, Joliette Alvarado‐Velásquez, Bárbara Ruiz‐Alayón, Cristina López‐Sánchez, José Ayala‐Hernández

**Affiliations:** ^1^ Grupo de Investigación Clínica Médica y Terapéutica A—Escuela Luis Razetti Universidad Central de Venezuela Caracas Venezuela; ^2^ Departamento de Medicina, Escuela de Medicina Luis Razetti Universidad Central de Venezuela Caracas Venezuela; ^3^ Servicio Medicina I Hospital Universitario de Caracas—Venezuela Caracas Venezuela

**Keywords:** brain stem infarction, chiropractic manipulation, pontine infarction, stroke, vertebral artery dissection

## Abstract

A 35‐year‐old woman with migraine and oral contraceptive use presented with pontine stroke and right vertebral artery occlusion following cervical manipulation by a lay practitioner. Managed conservatively outside the reperfusion window, she achieved progressive recovery. This case highlights a plausible temporal association between neck manipulation and ischemic events.

## Key Clinical Message

Cervical spine manipulation may be associated with vertebral artery occlusion and ischemic stroke, even in patients with pre‐existing vascular risk factors. Clinicians must maintain high suspicion when acute neurological deficits follow neck manipulation to ensure rapid diagnosis.

## 1 Introduction

Cervical manipulation therapy (CMT) is a technique frequently utilized in the management of various musculoskeletal disorders. However, it can lead to severe complications, including cervical artery dissection (CAD), which represents a significant etiology of ischemic stroke, particularly among young adults—accounting for up to 35% of stroke cases in this demographic [1].

The overall pooled incidence of stroke in Latin America and the Caribbean has been reported at 255 per 100,000 individuals. Currently, the global burden of stroke is predominantly concentrated in low‐ and middle‐income countries, which encompass over 65% of Latin American nations. Recent epidemiological data highlight an increasing burden of stroke in young individuals—termed “premature stroke”—representing 10% to 15% of all cases [2, 3]. Furthermore, posterior circulation strokes account for approximately 20% to 25% of all global ischemic strokes [4].

Thromboembolism is the most common pathophysiological mechanism of ischemic stroke secondary to CAD and correlates significantly with neuroimaging signs of thrombus formation [5]. More than 50% of CAD cases occur spontaneously and may be associated with underlying connective tissue disorders, such as fibromuscular dysplasia, Ehlers‐Danlos syndrome, Marfan syndrome, and Loeys‐Dietz syndrome [6]. Additionally, other conditions have been described, including α1‐antitrypsin deficiency, hyperhomocysteinemia, recent infections, vascular anatomical variations, history of migraine, and the use of oral contraceptives (OCs). Nevertheless, robust clinical literature has yet to confirm whether many of these variables represent definitive independent risk factors for arterial dissection [7].

Herein, we report the case of a 35‐year‐old female patient with a history of migraine and oral contraceptive use who developed a right vertebral artery occlusion accompanied by a pontine infarction following cervical spine manipulation by an unregulated lay practitioner. This report aims to provide clinical insights into the complex interaction between pre‐existing vascular risk factors and high‐velocity neck maneuvers, supporting a plausible temporal association rather than a definitive single etiology.

## 2 Case Presentation

### 2.1 Patient Information

A 35‐year‐old female patient with a 5‐year history of migraine without aura and a 3‐year history of oral contraceptive use (levonorgestrel 0.1 mg/ethinylestradiol 0.02 mg daily) presented to our institution. She had no personal history of hypertension, diabetes mellitus, or known hereditary thrombophilia, and her family history was non‐contributory.

Physical examination on admission revealed no somatic features suggestive of underlying connective tissue disorders or fibromuscular dysplasia. The patient exhibited normal stature and arm span, a negative Beighton score for joint hypermobility, no carotid bruits, normal baseline blood pressure, and no family history of vascular or aneurysmal disease.

### 2.2 Clinical Findings and Timeline

On February 20, 2025, following an acute stressful life event, the patient developed a severe, new‐onset holocranial, pressure‐type headache. Unlike her usual episodic migraines, which historically responded well to first‐line analgesics and ergotamines, this headache was continuous, atypical, and refractory to non‐steroidal anti‐inflammatory drugs and combination therapy (acetaminophen 650 mg, caffeine 50 mg, and dihydroergotamine 1 mg every 8 h).

Due to persistent symptoms, on February 26, 2025, she sought care for the first time at an informal outpatient community facility seeking pharmacological pain relief. Without a documented prior neurological examination, an unregulated lay practitioner performed a high‐velocity cervical spine manipulation. Immediately following the procedure, she experienced a sudden loss of consciousness lasting approximately 3 min, followed by complete spontaneous recovery. Upon awakening, she presented with acute right‐sided hemiparesis, right central facial palsy, and motor aphasia. Her blood pressure was recorded at 140/100 mmHg.

The patient was immediately transferred to our tertiary care center. Upon admission on February 26, 2025 (Day 0), an emergency non‐contrast brain computed tomography (CT) scan demonstrated no evidence of acute hemorrhage or established territorial infarction. On February 28, 2025 (Day 2), a non‐contrast brain magnetic resonance imaging (MRI) scan confirmed an acute ischemic lesion in the left pons (Figure 1).

**FIGURE 1:**
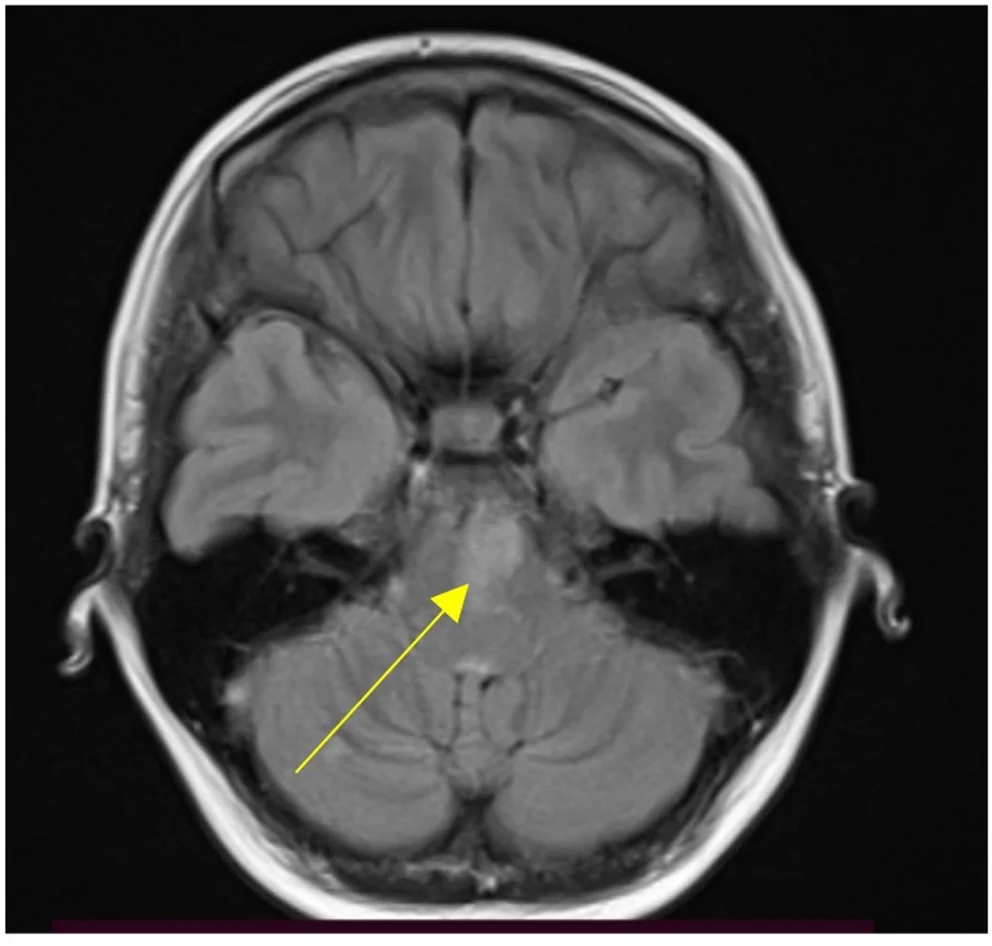
Axial T2‐FLAIR brain MRI demonstrating a hyperintense lesion in the left pons, consistent with an acute ischemic stroke (arrow).

### 2.3 Diagnostic Assessment

The brain MRI revealed hyperintensity on T2‐FLAIR and diffusion‐weighted imaging (DWI), with corresponding attenuation on the apparent diffusion coefficient (ADC) map in the left pons, consistent with an acute brainstem infarction (Figure 1).

Following the initial non‐contrast CT and MRI workup, and as allowed by institutional imaging logistics, a contrast‐enhanced head and neck CT angiography (CTA) was performed on March 7, 2025 (Day 9). The scan demonstrated a complete filling defect in the V1 to V3 segments of the right vertebral artery (RVA) and a thread‐like flow (“string sign”) in the V4 segment, with complete opacification of the basilar artery (Figure 2). In contrast, the left vertebral artery (LVA) remained fully patent with normal contrast filling.

**FIGURE 2:**
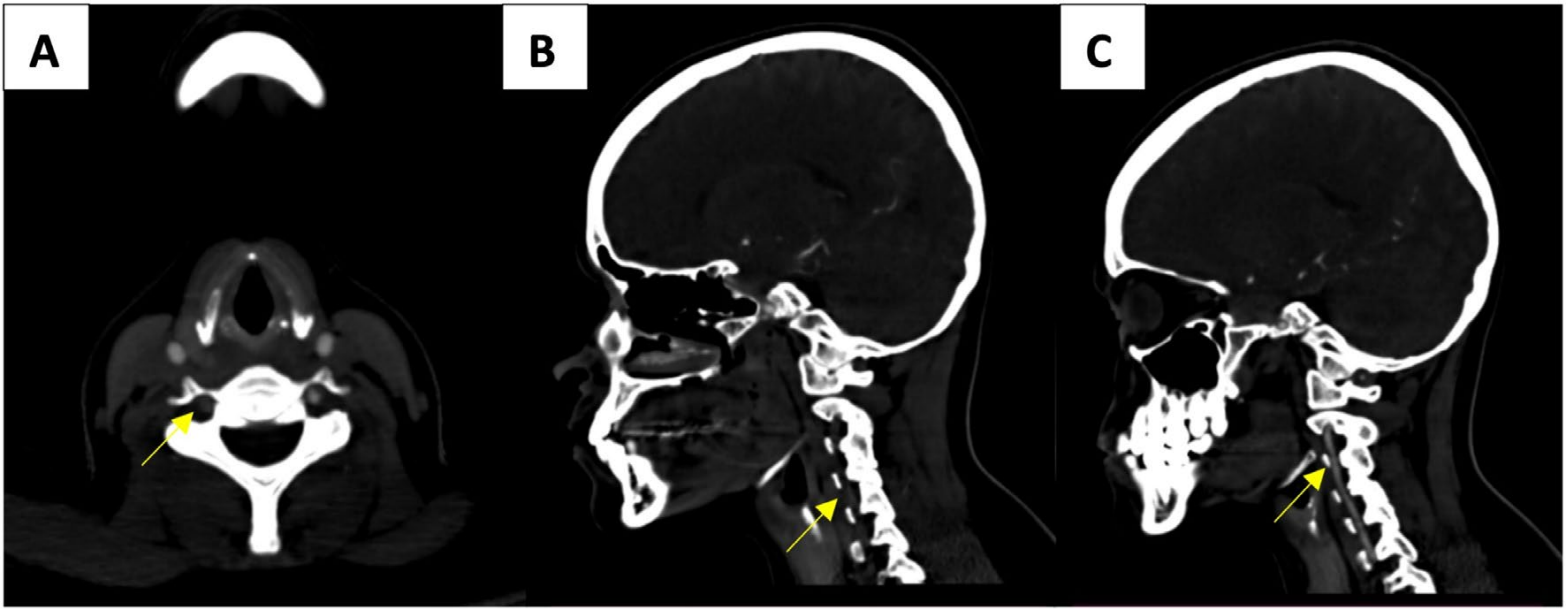
Head and neck CT angiography (CTA) with maximum intensity projection (MIP) reconstruction showing an arterial filling defect in the V1 through V3 segments of the right vertebral artery (RVA). (A) Axial view at the C6 vertebral level, demonstrating complete absence of contrast opacification in the RVA. (B) Sagittal view showing the non‐opacified RVA. (C) Sagittal view demonstrating a fully patent left vertebral artery (LVA) with normal contrast filling.

Although direct pathognomonic features of dissection—such as an explicit intimal flap, double lumen, or pseudoaneurysm—were not visualized on CTA, the segmental filling defect and distal string sign in a young patient without underlying atheromatous disease or thrombophilia strongly support a diagnosis of right vertebral artery occlusion highly compatible with vascular dissection. Other vascular abnormalities, including primary thrombosis and arterial hypoplasia, were considered in the differential diagnosis.

Based on the clinical timeline, neuroimaging findings, and exclusion of systemic thrombophilias, a final diagnosis of acute pontine ischemic stroke associated with right vertebral artery occlusion—highly compatible with arterial dissection—was established, with high‐velocity cervical manipulation serving as a potential triggering or contributing factor.

### 2.4 Treatment and Outcome

Upon admission to our tertiary center, the patient was beyond the therapeutic time window for reperfusion therapies, in addition to local institutional resource constraints. Therefore, medical management with single antiplatelet therapy (aspirin 81 mg daily) was initiated, guided by her NIHSS score, alongside early neurological rehabilitation. Following the implementation of this therapeutic regimen, the patient exhibited progressive resolution of her motor aphasia and partial improvement of her hemiparesis, although without complete recovery of her focal neurological deficit.

## 3 Discussion

High‐velocity thrust techniques used in cervical manipulation therapy (CMT) have been documented to place significant mechanical strain on the cervical vasculature [8]. Following arterial dissection, intramural thrombus accumulation or embolization substantially elevates the risk of acute ischemic stroke and severe neurological deficits [9].

In 2013, Wynd et al. conducted a systematic review evaluating the available evidence on cervical artery dissection (CAD) associated with CMT, applying the Bradford Hill criteria for causation to assess the strength of association between the exposure and the adverse event [10, 11]. Their findings demonstrated that approximately 70% of reported cases involved dissection of either the vertebral or carotid arteries. Within the subset of 707 cases reporting stroke type, 706 were ischemic, and among the 32 cases specifying anatomical location, the brainstem was the most common site (9 cases). Furthermore, 852 cases confirmed the diagnosis using digital subtraction angiography (34%), magnetic resonance imaging (MRI) with or without magnetic resonance angiography (34%), or computed tomography (CT) (9%). These epidemiological findings align closely with our patient's presentation regarding the ischemic stroke subtype, brainstem location, and neuroimaging modalities utilized.

Detection and diagnosis of CAD generally require a comprehensive neurological evaluation paired with advanced neuroimaging, such as head and neck CT angiography (CTA) or magnetic resonance angiography (MRA), with the latter recommended as the initial screening modality [12]. Nevertheless, variable sensitivity across imaging modalities has been described, with CTA demonstrating superior diagnostic accuracy for vertebral artery dissections specifically. This supports the diagnostic utility of the neuroimaging sequence performed in our case [13].

The hyperacute management of transient ischemic attack or acute ischemic stroke secondary to CAD does not differ from that of ischemic stroke from other etiologies, primarily relying on reperfusion therapies (intravenous thrombolysis or endovascular thrombectomy). Case reports have documented successful administration of recombinant tissue plasminogen activator (rtPA) followed by dual antiplatelet therapy, without bleeding complications during follow‐up [14]. Regrettably, resource limitations in our healthcare setting precluded the administration of reperfusion therapies; thus, conventional medical management for subacute ischemic stroke was initiated, focusing on secondary prevention with antiplatelet therapy [15].

The Cervical Artery Dissection in Stroke Study (CADISS) trial evaluated the comparative efficacy of antiplatelet agents versus anticoagulants for secondary stroke prevention in patients with carotid and vertebral artery dissections. Although constrained by sample size limitations due to the low event incidence, the trial demonstrated no statistically significant differences in stroke recurrence rates or angiographic recanalization rates between antiplatelet and anticoagulant regimens.

A crucial clinical consideration in this case is the 6‐day history of continuous, atypical holocranial headache before the cervical manipulation. In cervical artery dissections, severe localized or holocranial headache and neck pain frequently represent the primary prodromal symptom resulting from intramural nociceptive receptor stimulation before frank ischemic symptoms occur. It is highly plausible that arterial dissection or subclinical thrombosis was already in progress when the patient sought care, and the subsequent mechanical maneuver may have either exacerbated the vascular tear or dislodged a thromboembolism into the basilar circulation.

This clinical observation aligns with recent large‐scale epidemiological evidence. In a nationwide study of over 5000 cervical artery dissection cases, Whedon et al. (2023) demonstrated that patients undergoing evaluation and management for prodromal neck pain or headache were significantly more likely to develop subsequent dissection diagnoses than those receiving spinal manipulation. The authors concluded that the association between cervical manipulation and vertebral artery dissection is largely non‐causal, driven primarily by patients seeking symptomatic relief for an impending or active dissection. Furthermore, Whedon et al. emphasized that the absolute risk of vertebral artery dissection following cervical spine manipulation remains exceptionally low [9]. Our case underscores the importance of recognizing atypical prodromal headaches to avoid high‐risk manual interventions in patients with active arterial pathology.

## 4 Limitations

This report has limitations inherent to its single‐case retrospective design. First, while a close temporal association exists between cervical spine manipulation and the onset of acute neurological deficits, a definitive causal relationship cannot be established. Second, confounding predisposing factors—including oral contraceptive use, a history of migraine, and acute psychological stress—limit the attribution of the ischemic event solely to a mechanical trigger. Third, baseline pre‐event vascular imaging was unavailable for direct comparison, precluding definitive exclusion of pre‐existing arterial hypoplasia or vascular dysplasia before the acute episode.

## 5 Conclusion

The association between cervical manipulation therapy and cervical artery dissection has become a topic of growing clinical interest, as several studies suggest an increased relative risk; nevertheless, the overall absolute risk remains low given the rarity of the condition in the general population. However, this low incidence does not diminish its clinical significance, considering the potential for severe and permanent neurological sequelae.

This case highlights the critical need to maintain a high index of diagnostic suspicion when patients present with acute neurological symptoms following cervical manipulation, even in the absence of traditional cardiovascular risk factors. Prompt recognition enables timely neuroimaging evaluation and early initiation of appropriate medical management, which can substantially improve functional outcomes and reduce long‐term disability.

Because most current evidence linking cervical manipulation therapy to vertebrobasilar territorial stroke stems from case reports, case series, and expert opinion, improving the consistency, rigor, and thoroughness of reporting in such clinical encounters is essential. Enhanced reporting practices will help clarify the strength of this association and support the development of preventive strategies within the medical and scientific communities.

Clinicians must maintain a high index of suspicion when evaluating patients with severe, atypical, or refractory headaches before manual cervical therapies, as these symptoms may represent a prodromal arterial dissection in progress. Regulatory frameworks and public health awareness regarding unregulated cervical manipulation should be strengthened to prevent catastrophic cerebrovascular complications.

## Author Contributions

**Aguasanta González‐Delatorre:** investigation, conceptualization, writing – review and editing, writing – original draft. **Joliette Alvarado‐Velásquez:** conceptualization, writing – original draft, investigation, formal analysis. **Bárbara Ruiz‐Alayón:** investigation, validation, supervision. **Cristina López‐Sánchez:** supervision, investigation, writing – review and editing. **José Ayala‐Hernández:** conceptualization, writing – review and editing, supervision, data curation.

## Funding

The authors have nothing to report.

## Ethics Statement

The local institutional ethics committee waived formal approval for this case report in accordance with institutional policy, as this publication involves a single, non‐interventional, retrospective case description. The study was conducted in accordance with the Declaration of Helsinki.

## Consent

Written informed consent was obtained from the patient for the publication of this case report and any accompanying clinical images.

## Conflicts of Interest

The authors declare no conflicts of interest.

## Data Availability

The data that support the findings of this study are available from the corresponding author upon reasonable request.
